# Double Down on Double Vision: An Unusual Case of Painful Diplopia

**DOI:** 10.7759/cureus.20838

**Published:** 2021-12-30

**Authors:** John Oghene, Sri Harsha Boppana, Pooja Reddy, Bryce D Beutler, Deepan Dalal

**Affiliations:** 1 Department of Internal Medicine and Primary Care, Brown University, Warren Alpert Medical School, Providence, USA; 2 Department of Internal Medicine, University of Nevada, Reno School of Medicine, Reno, USA; 3 Department of Rheumatology, Brown University, Warren Alpert Medical School, Providence, USA; 4 Department of Radiology, University of Southern California, Keck School of Medicine, Los Angeles, USA

**Keywords:** tolosa-hunt syndrome, ophthalmoplegia, wegener granulomatosis, granulomatosis with polyangiiitis, autoimmune disease

## Abstract

Tolosa-Hunt syndrome (THS) is a rare neuro-immunological disorder characterized by severe periorbital headaches and ophthalmoplegia. In some patients, THS may occur in parallel with other autoimmune disorders. The underlying etiology of THS remains to be definitively established. However, inflammation of the cavernous sinus or orbital apex represents a hallmark feature; magnetic resonance imaging, therefore, plays a key role in establishing a diagnosis. We describe a patient who presented with concomitant THS and granulomatosis with polyangiitis. In addition, we describe the clinical and imaging findings of THS and review treatment options for this rare condition.

## Introduction

Tolosa-Hunt syndrome (THS) is one of the rare neuro-immunological diseases that typically occurs in late middle age, most commonly presenting in the seventh decade of life. The estimated incidence of Tolosa-Hunt syndrome is about one patient per million in a year and has been recognized by the National Organization for Rare Disorders as a cause for headaches [1,2]. In 1954, Dr. Eduardo Tolosa, a Spanish neurosurgeon, first described this painful benign cause of ophthalmoplegia. Clinical features of THS include the acute onset of severe periorbital headache followed by painful and restricted eye movements as well as double vision and paralysis of one or more of the third, fourth, and/or sixth cranial nerves. Magnetic resonance imaging (MRI) typically demonstrates inflammatory changes in the anterior cavernous sinus, superior orbital fissure, or the apex. THS is characterized by inflammation of the cavernous sinus of the brain or orbital apex, with resultant cranial nerve palsy. Although an underlying cause of THS remains to be established, it is favored to be autoimmune in etiology given its strong association with systemic lupus erythematosus and other immune-related conditions [3-6]. There is no known sex or age predilection [7,8]. Numerous cases are reported in the literature. However, there is limited information highlighting the association between THS and granulomatosis with polyangiitis (formerly Wegner granulomatosis), a type of vasculitis affecting blood vessels in sinuses, lungs, and kidneys with associated neurological complications including seizures, focal motor/sensory complaints, and stroke [9]. We present a case of this syndrome in association with granulomatosis with polyangiitis which was successfully managed with a high dose of corticosteroids and rituximab infusion.

## Case presentation

**Figure 1:**
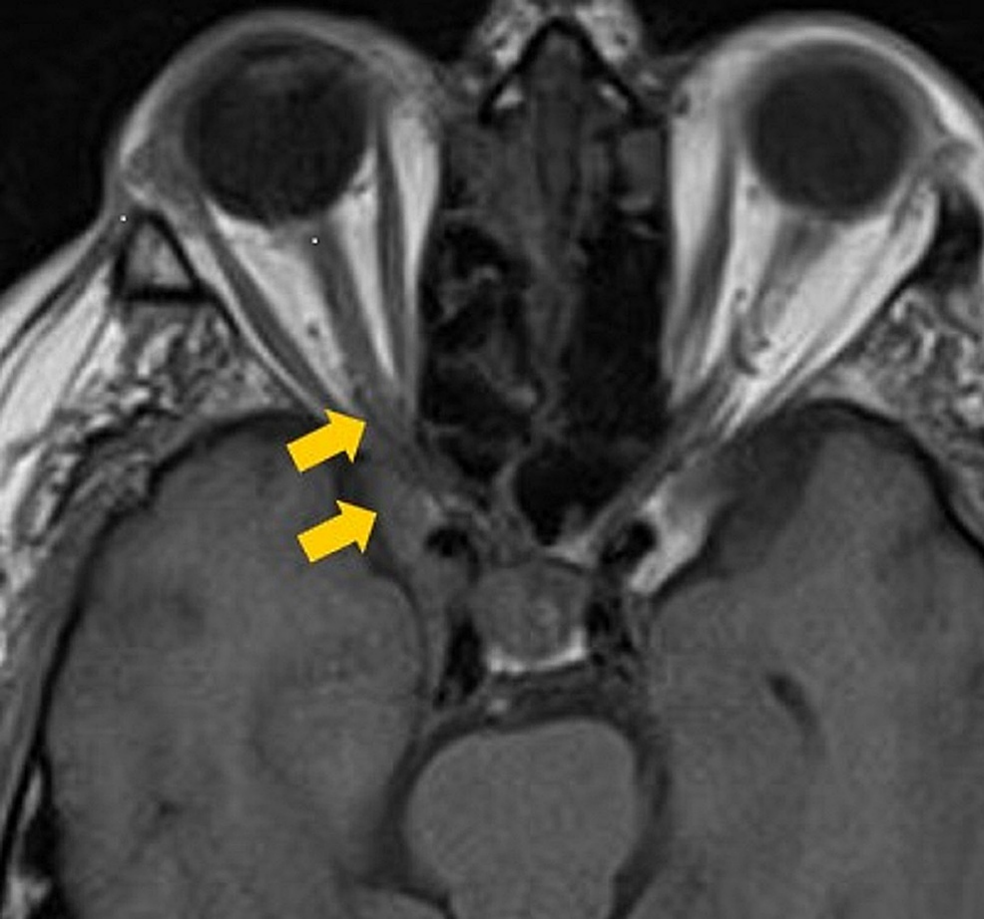
Initial axial pre-contrast T1-weighted MRI The image is demonstrating inflammatory changes in the right posterior orbital apex (yellow arrow) consistent with Tolosa-Hunt syndrome.

**Figure 2:**
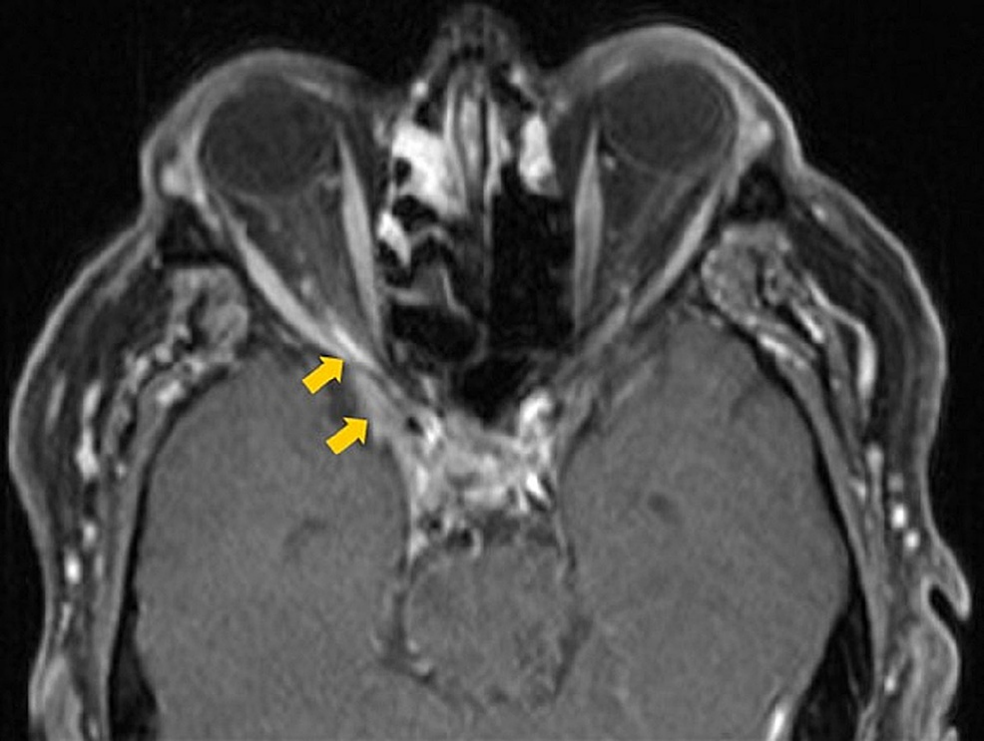
Initial axial contrast-enhanced T1-weighted MRI The image is demonstrating enhancement of the right posterior orbital apex (yellow arrow).

**Figure 3:**
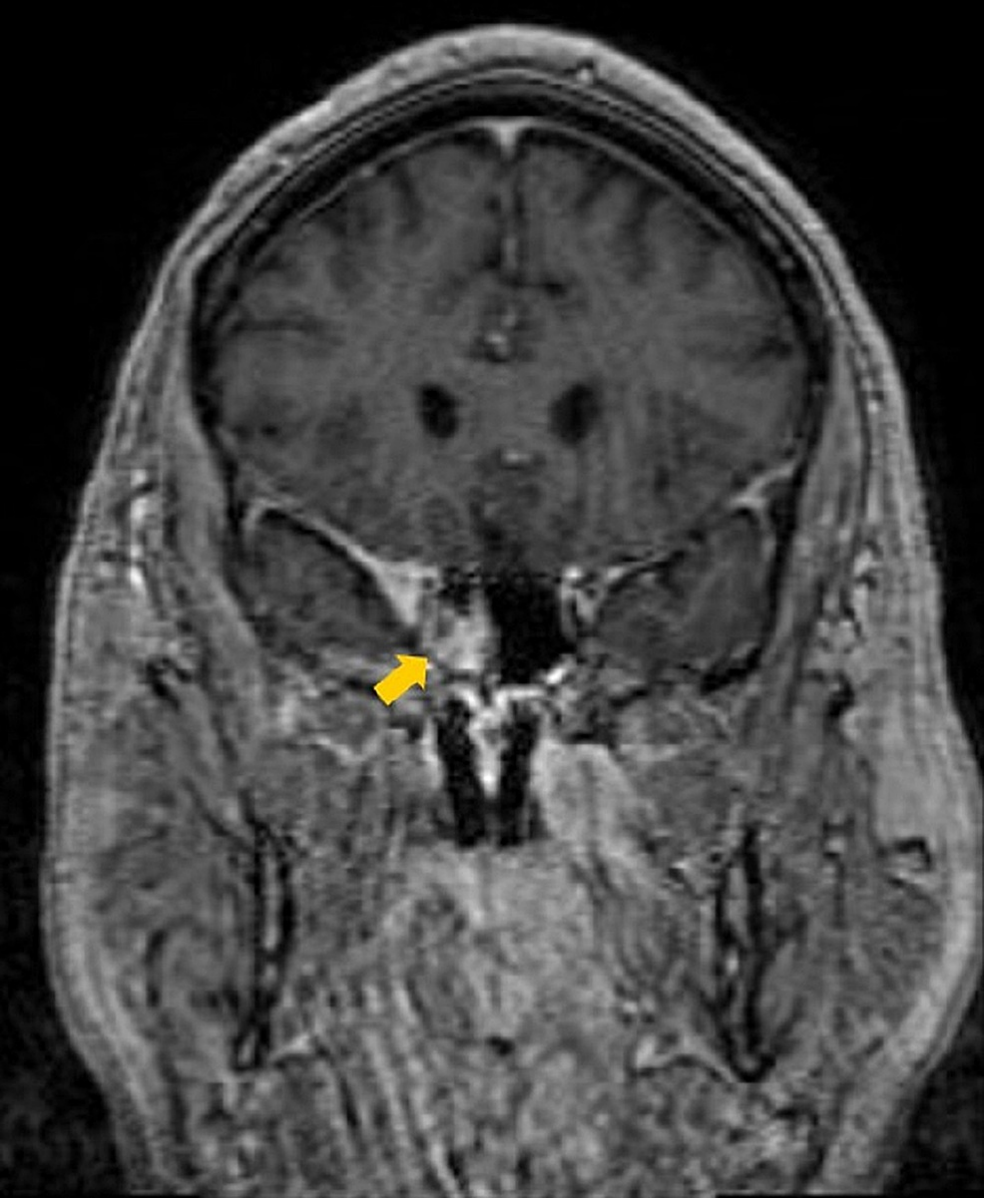
Initial coronal contrast-enhanced T1-weighted MRI The image is demonstrating enhancement of the right cavernous sinus extending into the right orbital apex (yellow arrow).

A 60-year-old male with recurrent chronic sinusitis of one-year duration presented to the emergency room for evaluation of the four-week history of progressive stabbing right eye pain that radiated to the back of his eye. The patient reported that he had also developed double vision two days after the initial onset of the eye pain. The initial neurologic examination was significant for diplopia on the right gaze. There was no exophthalmos. Intraocular pressure was normal. Afferent visual function - including visual acuity, color vision, pupillary responses, and automated visual field testing - was also normal. A dilated fundoscopic examination showed healthy-appearing optic nerves without edema or pallor. Initial imaging - including CT scan of the head, CTA of the head and neck, EKG, chest x-ray, and echocardiogram - showed no evidence of acute intracranial hemorrhage or mass effect. Contrast-enhanced T1-weighted MRI of the brain showed enhancement of the right cavernous sinus extending into the right orbital apex as well as mild thickening and enhancement of the right extraocular muscles (Figures 1-3).

There was diffuse hyperintensity of the right cavernous sinus on T2-weighted sequences. CT chest revealed a small spiculated right apical lung nodule and patchy ground-glass opacities in both lungs. Laboratory studies revealed a positive antinuclear antibody (ANA), an elevated cytoplasmic antineutrophil cytoplasmic antibody (c-ANCA), and anti-proteinase-3 antibodies, which are highly specific for granulomatosis with polyangiitis in the appropriate clinical context. All other laboratory testing was within normal limits (Table 1).

**Table 1:** Initial laboratory studies ESR: erythrocyte sedimentation rate; CRP: C-reactive protein; CBC: complete blood count; CMP: cardiomyopathy; TSH: thyroid-stimulating hormone; ANA: antinuclear antibody; c-ANCA: cytoplasmic antineutrophil cytoplasmic antibody; p-ANCA: perinuclear antineutrophil cytoplasmic antibody; RPR: rapid plasma regain; SPEP: serum protein electrophoresis; Ab: antibody; ACE: angiotensin-converting enzyme; CSF: cerebrospinal fluid; FTA: fluorescent treponemal antibody; hepatitis B core/s Ag: hepatitis B core/surface antigen

Laboratory Study	Result	Reference Range
ESR	20 mm/h	0.0-20 mm/h
CRP	2.53 mg/L	0.0-10.0 mg/L
CBC	Normal	Normal
CMP	Normal	Normal
TSH	1.213 ulU/mL	0.350-5.500 ulU/mL
ANA	1:80	< 1:40
c-ANCA	1:160	< 1:20
p-ANCA	< 1:20	< 1:20
Proteinase-3 antibody	> 8.0	0.0-0.9
dsDNA Ab	< 1 IU/mL	0.0-4 IU/mL
Lyme Ab	< 0.2	0.0-0.8
RPR/FTA	Non-reactive	Non-reactive
SPEP	Normal	Normal
IgG panel	Normal	Normal
Quant-gold	Negative	Negative
Hepatitis B core/s Ag	Non-reactive	Non-reactive
Hepatitis C Ab	Non-reactive	Non-reactive
ACE level	30 U/L	9-67 U/L
CSF analysis	Normal	Normal

Based on the clinical presentation, laboratory, and imaging results of the patient, a presumptive diagnosis of THS and granulomatosis with polyangiitis was established. The case was discussed with the neuro-ophthalmology, pulmonology, and rheumatology teams, who recommended a trial of immunosuppressant medication rather than attempting biopsy or cerebrospinal fluid analysis to confirm the diagnosis. Initial treatment was started with prednisone 60 mg daily; orbital pain improved within 48 hours. Prednisone was then tapered and the patient was started on weekly infusions of rituximab at 250 mg every four weeks. After three months of treatment, the patient was symptom-free. Follow-up laboratory studies revealed minimally elevated erythrocyte sedimentation rate (ESR) and normal C-reactive protein (CRP) (Table 2).

**Table 2:** Follow-up laboratory studies of three months ESR: erythrocyte sedimentation rate; CRP: C-reactive protein; CBC: complete blood count; CMP: cardiomyopathy; TSH: thyroid-stimulating hormone

Laboratory Study	Result	Reference Range
ESR	22 mm/h	0.0-20 mm/h
CRP	1.42 mg/L	0.0-10.0 mg/L
CBC	Within normal	Normal
CMP	Within normal	Normal
TSH	1.147 ulU/mL	0.350-5.500 ulU/mL

**Figure 4:**
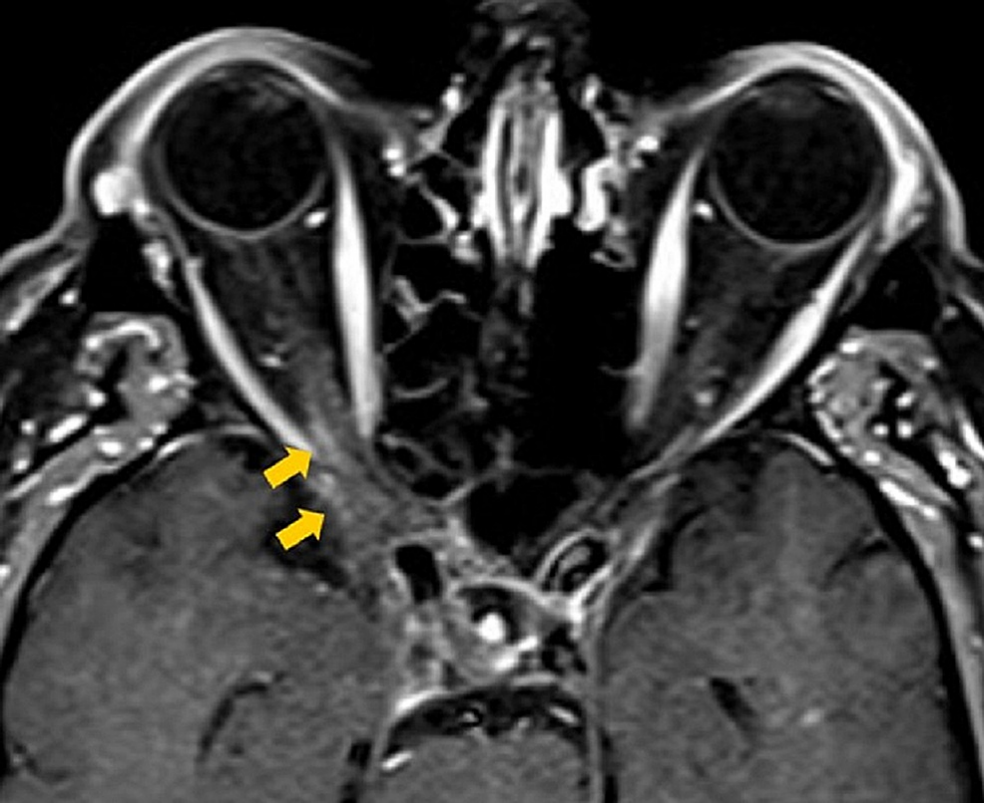
Three-month follow-up axial contrast-enhanced T1-weighted MRI The image is showing markedly decreased inflammation of the right posterior orbital apex and right cavernous sinus (yellow arrows) after treatment with prednisone 60 mg.

**Figure 5:**
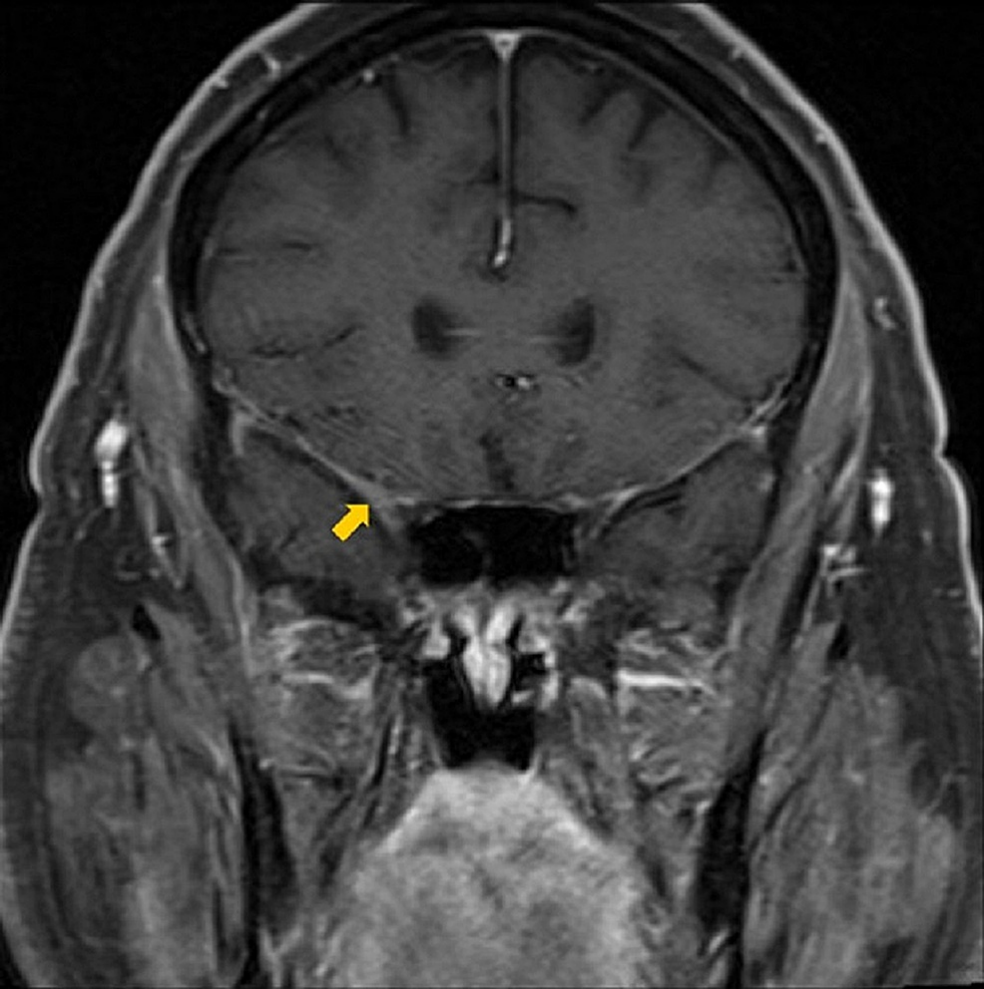
Three-month follow-up coronal contrast-enhanced T1-weighted MRI The image is showing markedly decreased inflammation of the right posterior orbital apex and right cavernous sinus (yellow arrows) after treatment with prednisone 60 mg.

Repeat MRI orbit showed persistent but markedly decreased T2 hyperintensity of the cavernous sinus near the posterior orbital apex on the right (Figures 4, 5). The patient remains asymptomatic on 250 mg rituximab infusions every six months.

## Discussion

Tolosa-Hunt syndrome is a rare cause of ophthalmoplegia associated with pain which quickly responds to steroids by reversing the inflammation of cavernous sinus and orbital apex as demonstrated by repeat imaging [10]. The etiology of this disease falls into three broad categories including vascular, neoplastic, and inflammatory conditions. To diagnose Tolosa-Hunt Syndrome, physicians mainly rely on magnetic resonance imaging and its response to immunosuppressive treatment after ruling out all other causes of this symptomatology [11-13]. In this patient, his ANA, c-ANCA, and anti-proteinase-3 antibodies were positive suggesting the diagnosis secondary to a vasculitis such as granulomatosis with polyangiitis; an association that has been described in previous literature reviews. Immunosuppression with steroid therapy has been the main course of treatment with the resolution of findings on repeat imaging studies in these patients [14]. However, the difference in response time for full recovery of paralysis of cranial nerve among patients started on steroid therapy makes treatment challenging [12]. There has been literature on using other immunosuppressants like methotrexate, infliximab, rituximab, and radiotherapy to treat this debilitating syndrome [15-17]. In addition, B lymphocytes have been shown to play a significant role in the pathogenesis of ANCA-associated vasculitides. Rituximab, an anti-cluster of differentiation antigen 20 (anti-CD20) monoclonal antibody, decreases B lymphocyte activity [18]; this agent is now used for the management of ANCA-associated vasculitis [19].

A diagnosis of THS can usually be established through correlation of clinical presentation, laboratory findings, and contrast-enhanced brain and orbit MRI features. Positive serologies in the setting of classic clinical features have a very high positive predictive value. PET/CT may also be used for diagnosis and monitoring [20]. In rare cases, a tissue biopsy may be considered to confirm the diagnosis. However, in many individuals, including our patient, a biopsy is technically challenging and can be avoided if the clinical symptomatology, laboratory findings, and neuroimaging are compatible with THS.

## Conclusions

Although Tolosa-Hunt syndrome is one of the causes of painful ophthalmoplegia, recognizing the symptoms early enough to treat will increase the symptom-free time. There is no definite consensus on the treatment approach, but treatment with steroids is widely accepted along with the option of various immunosuppressants, such as methotrexate, infliximab, and rituximab. Our patient was treated with a combination of steroids and rituximab resulting in relief of symptoms. There needs to be more research on the diagnostic criteria and treatment guidelines to prevent the recurrence of these symptoms.
